# Cylinder Filling

**Published:** 1880-07

**Authors:** H. B. Spencer


### CYLINDER FILLING.

BY H. B. SPENCER.

*Read before the Tenn. State Dental Society.*

Who first filled teeth with cylinders I have not been able to learn, but presume the practice is almost as ancient as the use of gold for filling in any other form. In order to arrest the ravages of decay in teeth with any filling material it is necessary to accomplish two things perfectly.

1st. Every particle of decay must be removed.

2d. The cavity must be filled in such a manner as to exclude entirely the fluids of the mouth from its inner part.

This is accomplished in various ways by different operators. I shall in this paper try to describe one method only, which has proved reasonably successful in my hands. This method consists in what is usually termed " cylinder filling." The cylinders are generally made of No. 6 soft foil. The foil is first crimped, and if the cavity is a medium sized one, it is cut or torn into four equal parts, then with a small bladed spatula, these parts are folded into strips the width of which corresponds with the depth of the cavity to be filled. With a pair of sharp pointed pliers, such as are generally used by jewelers, these strips are rolled into cylinders of different sizes. Some of the smaller ones are made more pointed by grasping them diagonally at one end, and with the rolling pliers, giving them an extra fold; others are flattened and pressed into wedge shape. These are very important factors in cylinder filling. The cavity if it be in the crown of a molar tooth is prepared by making at least two under cuts as nearly opposite each other as practicable; then a cylinder, the diameter of which is almost as great as that of the cavity, is pressed to one of the walls containing the undercut, another is pressed to the opposite wall, then the intervening space is filled with smaller cylinders. After the cavity is full it is necessary to test its solidity with a needle shaped point, and whenever permeations can be made, sharp pointed cylinders are introduced.

The mass can then be condensed by hand pressure, or with the mallet. The cylinders having been placed in the cavity, parallel with its depth, it will be seen that the finish will be upon the edge of the foil, so there will be no danger of scaling or tearing at the margins. A crown filling belonging to the class of the one above described, is simple and the operation is easily accomplished. The other extreme is reached when we attempt to fill the proximal surfaces of the incisors where the palatel and labial walls are broken away. This is done by first making concavities or undercuts in the cervical and opposite walls. The cylinders to be used should be pressed and flattened, and should be of sufficient width when placed in the cavity to extend from the palatel to to the labial wall. The first cylinder is placed against the cervical wall, and with a foot shaped plugger, pressed sufficiently to make it remain in position, while another is pressed against the opposite wall with a curved point. Then the space intervening is filled in the same way, with cylinders flattened and wedge-shaped. The cavity should be filled full enough to admit of an oval finish, especially on the lip surface. There are other cavities, such as those found in the proximal surfaces of the bicuspids and molars, which require great care, skill and patience to treat succesfully. The general rules to be observed in filling them are the same as those in the above described operations, with variations of course in proportion to the difference in shape of cavity, location, &c. The foil is crimped before being rolled into cylinders for the purpose of giving it pliability. Soft foil is preferred for the reason that it readily conforms to all irregular wall surfaces, and consequently prevents leakage.

Dr. A. Westcott, formerly professor of operative dentistry in the Baltimore College of Dental Surgery and late professor in the New York College of Dental Surgery, claims to have been the first to discover, or at least to utilize the adhesive or cohesive qualities of gold foil, in the year 1840. In the year 1870, thirty years after the date of this discovery, in an article published in the Dental Cosmos, he says, "While I condemn no one's method, I may be allowed to prefer one practice as having, in my judgment, advantages over others. Formerly, or in the early stages of my practice with adhesive foil, I used it to fill the entire cavity, whether great or small, but now make the following modifications:

1st. I seldom or never now fill a cavity entirely with adhesive foil.

2d. I now use from eight to ten times as much soft foil, on the average, as I do adhesive foil.

3d. I uniformly use my soft foil in the form of cylinders, varying in length, diameter and compactness to suit the circumstances of the case.

4th. After the operation is carried as far as it can be with certainty, with cylinders, I then use firm but small pointed pluggers, with but little taper, and pierce any and every part of the filling, then treat these permeations as new or original cavities. These I fill with adhesive foil, *and this is now the only way or manner in which I use adhesive foil.*"

This coming as it does from one who had thoroughly tested gold as a filling material in all its varied forms and conditions for nearly a third of a century, is certainly worth remembering.

I do not wish to make the impression on any one by what I have said in this brief paper, that I discourage or disapprove the use of cohesive gold. There are circumstances under which no other kind of gold can be used to make a durable filling.

